# High-Performance MIM Capacitors for a Secondary Power Supply Application

**DOI:** 10.3390/mi9020069

**Published:** 2018-02-04

**Authors:** Jiliang Mu, Xiujian Chou, Zongmin Ma, Jian He, Jijun Xiong

**Affiliations:** 1 Key Laboratory of Instrumentation Science and Dynamic Measurement, Ministry of Education, North University of China, Taiyuan 030051, China; mzmncit@nuc.edu.cn (Z.M.); drhejian@nuc.edu.cn (J.H.); xiongjijun@tsinghua.org.cn (J.X.); 2 School of Instrument and Electronics, North University of China, Taiyuan 030051, China

**Keywords:** microelectromechanical systems (MEMS), microstructures, metal-insulator-metal capacitors, secondary power supply, electrical properties

## Abstract

Microstructure is important to the development of energy devices with high performance. In this work, a three-dimensional Si-based metal-insulator-metal (MIM) capacitor has been reported, which is fabricated by microelectromechanical systems (MEMS) technology. Area enlargement is achieved by forming deep trenches in a silicon substrate using the deep reactive ion etching method. The results indicate that an area of 2.45 × 10^3^ mm^2^ can be realized in the deep trench structure with a high aspect ratio of 30:1. Subsequently, a dielectric Al_2_O_3_ layer and electrode W/TiN layers are deposited by atomic layer deposition. The obtained capacitor has superior performance, such as a high breakdown voltage (34.1 V), a moderate energy density (≥1.23 mJ/cm^2^) per unit planar area, a high breakdown electric field (6.1 ± 0.1 MV/cm), a low leakage current (10^−7^ A/cm^2^ at 22.5 V), and a low quadratic voltage coefficient of capacitance (VCC) (≤63.1 ppm/V^2^). In addition, the device’s performance has been theoretically examined. The results show that the high energy supply and small leakage current can be attributed to the Poole–Frenkel emission in the high-field region and the trap-assisted tunneling in the low-field region. The reported capacitor has potential application as a secondary power supply.

## 1. Introduction

Metal-Insulator-Metal (MIM) capacitors, which are typical passive components, have been widely used for radio-frequency decoupling and analog mixed signal integrated circuits applications [1,2,3,4,5,6,7,8] due to their low parasitic capacitance and low resistivity electrode [6,9]. Also, MIM capacitors are attractive energy storage devices and can act as a secondary power supply due to the excellent advantage of their rapid-moving charge and high burst power [10].

A typical application for a secondary power supply is as an ignition device, for which some key parameters, including moderate energy density and excellent power density combined with high capacitance density, a high breakdown voltage, and a low leakage current, are required. Generally, energy density, which is usually referred to as areal energy density, is proportional to capacitance density and the square of breakdown voltage. It is clear that high capacitance density can be achieved by using a high-*k* dielectric and low dielectric thickness according to the operating principle of planar capacitors. In contrast, this way can result in a low breakdown voltage according to the empirical relation [11] and the electric field strength equation. It seems that there is an established inherent tradeoff between capacitance density and breakdown voltage. However, the high energy density of a MIM capacitor can only be achieved by using a high dielectric thickness [12] due to the coupling effect of thickness on capacitance density and breakdown voltage.

In some secondary power supply applications, the footprint area of the capacitors is limited. Under these conditions, the energy density per unit planar area instead of the areal energy density is an exact evaluation criterion for MIM capacitors. Hence, the question of how to increase the specific surface area based on fixed appearance sizes becomes particularly important. Concerning this, many researchers have made much effort to fabricate three-dimensional (3D) structures by anodic aluminum oxide macro-holes [10] or silicon micro-holes [13], in which large areal MIM layers are grown. So far, more attention has been paid to high-*k* dielectrics, including HfO_2_ [1,8], Al_2_O_3_ [2,14], TaYO*_x_* [3], ZrO_2_ [4], Lu_2_O_3_ [6], and Eu_2_O_3_ [15] and their combinations, such as sandwiched [9,16], stacked [5,7], and laminate structures [17,18], based on the substrate areas of within the millimeter scale in the past few years. Currently, most of the 3D MIM capacitors are being used for dynamic random access memory. Specifically, a flexible 3D MIM capacitor on silicon fabric has been successfully fabricated and experimentally demonstrated by Hussain et al. [19,20,21], which is very innovative about the manufacturing process and is very promising for flexible electronics. In these capacitors, the working voltages are mostly around the 10 V used for integrated circuits. However, a high working voltage is essential for MIM capacitors with a large specific surface area to meet the requirement of a secondary power supply, where the typical voltages range from 20 V to 30 V. It is noticeable that keeping a high breakdown voltage is difficult for a large areal MIM capacitor, because large areas make the probability of defective dielectrics greatly increase and easily induce a large leakage current, which is the origin of the premature breakdown of MIM capacitors. So, an effective way is to use a large dielectric thickness. Still, high-*k* dielectrics would be necessary to compensate for the reduced capacity induced by a thick dielectric [12]. In this work, to meet the requirement of a secondary power supply application, the planar area and 3D area of the MIM capacitors are designed to be 100 mm^2^ and 2.45 × 10^3^ mm^2^, respectively. To the best of our knowledge, the two values are much larger than the previously reported ones. Therefore, it is necessary to systematically investigate the MIM capacitors’ performance.

In this present paper, we successfully fabricated high-performance MIM capacitors with a thick Al_2_O_3_ dielectric on high aspect ratio substrates. To evaluate the electrical properties of the MIM capacitors, the capacitance’s dependence on voltage and frequency, the conduction mechanism, and the energy density are investigated experimentally. We found that the fabricated MIM capacitors show a large breakdown voltage, a low leakage current, moderate energy density, and small capacitance variation, which largely benefit secondary power supply applications.

## 2. Materials and Methods

**Figure 1:**
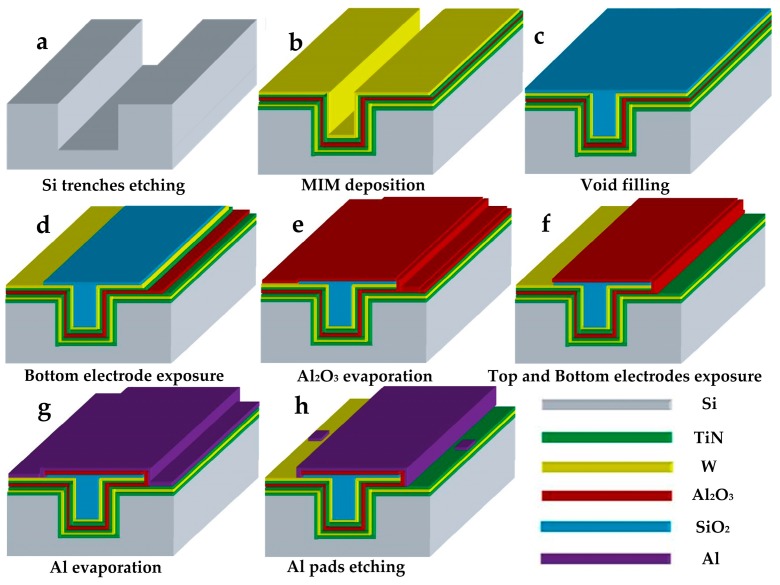
Process sequence to fabricate metal-insulator-metal (MIM) capacitors. (**a**) Si trenches etching, (**b**) MIM deposition, (**c**) Void filling, (**d**) Bottom electrode exposure, (**e**) Al_2_O_3_ evaporation, (**f**) Top and Bottom electrodes exposure, (**g**) Al evaporation, (**h**) Al pad etching.

In our study, one 8 inch, 750 μm thick, double-side polished (100) *n*-type silicon wafer is designed as 224 square cells with the size of 10 mm × 10 mm. Figure 1 exhibits the process sequence to fabricate the MIM capacitors. Firstly, the wafer is etched by the deep reactive ion etching process into micro-trench arrays with a width of 6 μm and a depth of 160 μm as shown in Figure 1a. The capacitors are formed on high aspect ratio micro-trench structures that we have previously reported [22]. Then, 300 nm SiO_2_ is grown by dry oxidation as an isolation layer to avoid the premature breakdown of the MIM capacitors due to the rough sidewall surface of the trenches. In Figure 1b, the electrode films of W/TiN and the dielectric film of Al_2_O_3_ are deposited by atomic layer deposition with the precursors WF_6_/SiH_4_, Al(CH_3_)_3_/H_2_O, and TiCl_4_/NH_3_ at 300 °C, respectively. The resulting MIM capacitors are comprised of the stack of TiN (10 nm)/W (50 nm)/TiN (10 nm)/Al_2_O_3_ (40 nm and 55 nm)/TiN (10 nm)/W (50 nm). Next, the void of the trenches is filled and smoothed with chemically evaporated SiO_2_, which acts as a chemical passivation layer for the top electrode as shown in Figure 1c. Subsequently, conventional lithography steps are employed to create the top and the bottom electrodes of the MIM capacitors as shown in Figure 1d–f. In Figure 1g–h, an Al film with the thickness of 1 μm is evaporated and then patterned to produce Al pads for the back-end wire bonding and electrical testing of the capacitor device.

The morphologies of the MIM capacitors were characterized using a Hitachi S-5500 (Tokyo, Japan) scanning electron microscope (SEM). The *C*–*V* curves on different frequencies and the *J*–*V* curves of the MIM capacitors were obtained using an Agilent 4284A (Santa Clara, CA, USA) and a Keithley 4200SCS (Cleveland, OH, USA), respectively. All of the measurements were carried out at room temperature.

## 3. Results and Discussion

### 3.1. Structural Morphologies

**Figure 2:**
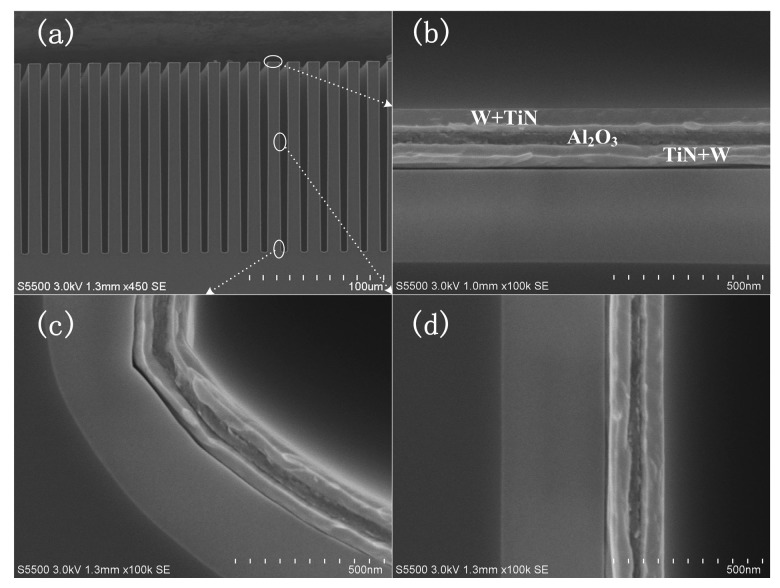
Cross-sectional SEM images of MIM capacitor A. (**a**) the overview images, (**b**) trench top, (**c**) trench bottom, (**d**) trench sidewall.

In our study, two kinds of MIM capacitors with the dielectric thickness of 40 nm and 55 nm are fabricated, and are labeled as capacitor A and capacitor B, respectively. Figure 2a shows the overview cross-sectional SEM images of capacitor A with the as-deposited electrodes and dielectric layers, while Figure 2b–d present the close-up view images of the top, bottom, and sidewall of the capacitor marked in circles in Figure 2a. The thicknesses of the Al_2_O_3_ are 40.5 nm, 40.2 nm, and 39.7 nm in Figure 2b–d, respectively. Also, the MIM capacitors exhibit clear boundaries at the interfaces between different layers. This indicates that large areal MIM capacitors were obtained on large areal high aspect ratio structures combined with the atomic layer deposition process.

### 3.2. Frequency Characteristics

**Figure 3:**
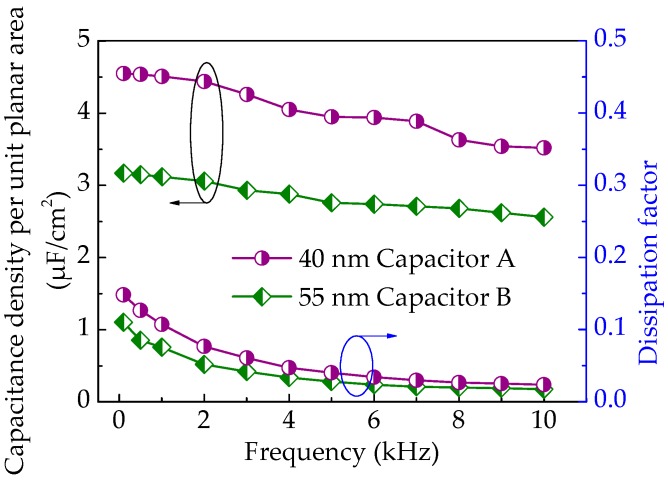
Capacitance density per unit planar area and dissipation factor with frequency of capacitors A and B.

To investigate the frequency characteristics, capacitors A and B were measured using 4284A and 4200SCS. Figure 3 shows the capacitance density per unit planar area (*C*) and dissipation factor (tan*δ*) with applied frequency (*f*) for capacitors A (the circle line) and B (the triangle line). It is found that the capacitance densities of both capacitors exhibit a slight degradation with increasing frequencies in the range of 1 kHz to 10 kHz. This indicates that frequencies higher than 100 Hz result in notable dispersion, which is supported by the report [10]. This phenomenon reflects a middle and high frequency dispersion feature of the dielectric and indicates the formation of stronger dipolar polarization [23]. Further, the dependence of capacitance density per unit planar area with an applied frequency can be expressed by the following equation [3]:

(1) $$C=C_{m}\left(1+\frac{A}{1+{\left(f/f_{c}\right)}^{2n}}\right)$$

where the bracket item at the right of the equation represents the non-linearity factor, *f_c_* is the cutoff frequency and equals 1/2*πτ* (*τ* denotes the relaxation time constant of the dielectric), *C_m_* represents the bulk-related capacitance (*f >> f_c_*), *n* is a value in the range of 0 to 1, and *A* is an amplitude factor. When the values of *f_c_* and *C_m_* are constant, the non-linearity factor decreases with increasing applied frequency. Thus, the resulting capacitance density per unit planar area decreases. In addition, the capacitance *C* is defined as *dq*/*dt* = *C dv*/*dt*, where the left item and the right second one of the equation denote the rate of change in the charge and voltage in the measurement setup, respectively. Compared with a planar capacitor, 3D capacitors with high aspect ratio structures have complex morphologies, such as edges, corners, and sidewall spikes, resulting from the deep reactive ion etching process. These irregular morphologies easily cause localization of an enhanced electric field, which might impede the response of charge. Due to the carriers’ inability under a fast-changing voltage, *dv*/*dt* is unable to follow a higher frequency and then *C* has to drop [20,21].

In this work, a series equivalent circuit model was used. For the dissipation factor, it is observed from Figure 3 that capacitors A and B present a decline from 0.107 to 0.023 and from 0.075 to 0.017 towards a higher frequency, and have a minimum average value 0.047 and 0.033 in the frequency range of 1 kHz to 10 kHz, respectively. The dispersion of dielectric loss can be calculated by the following equation [8,21]:

(2) $$G_{s}=D\omega C_{s}$$

where *G_s_*, *C_S_*, and *D* are the conductance, series capacitance, and dissipation factor, respectively, and ω equals 2π*f*. As can be seen, the dissipation factor decreases with increasing frequency and is frequency-dependent.

**Figure 4:**
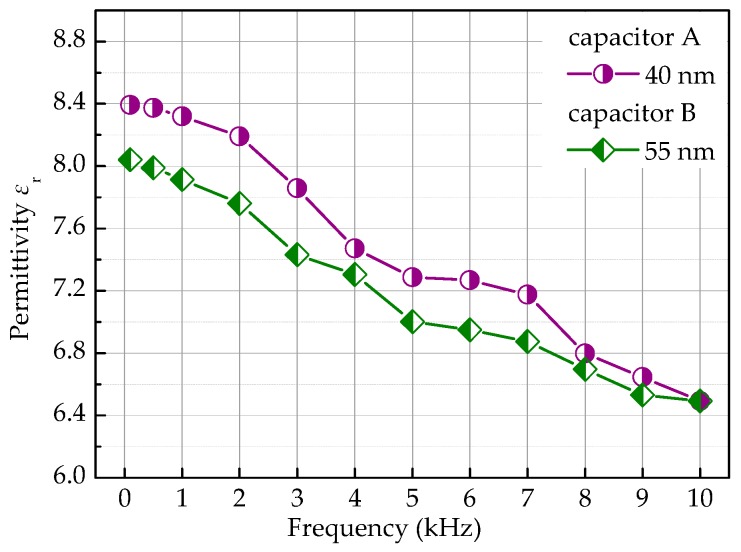
Dependencies of permittivity on frequency for capacitors A and B.

Figure 4 depicts the dependencies of permittivity (*ε_r_*) on frequency for capacitors A and B. In this figure, the circle line and the triangle line represent the *ε_r_*–*f* curves of capacitor A and capacitor B, respectively. It is observed that the permittivity of the two capacitors decreases as the frequency increases ranging from 1 kHz to 10 kHz. This result indicates that the MIM capacitors have frequency dispersion, especially in the range of middle and high frequency. The frequency dispersion of the fabricated MIM capacitors is attributed to the universal dielectric response.

Generally, capacitors for energy and high-power applications operate in the dozens of Hertz only [10]. Therefore, we would like to emphasize the performance of the capacitors in the lower frequency region. In this contribution, the average permittivity of capacitors A and B is approximately 8.2 at 100 Hz. The result is close to the bulk level *ε_r_* = 8~9, and is comparable with other planar capacitors’ reported values of *ε_r_* = 9 for 100 nm [12] and *ε_r_* = 8.7 for 40 nm [14].

### 3.3. J–V Characteristics

**Figure 5:**
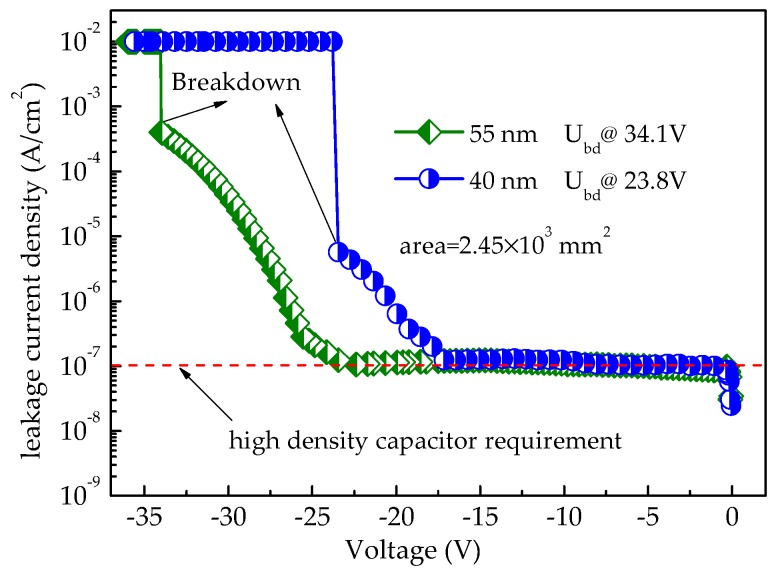
The leakage current density’s dependence on the voltage for capacitors A and B.

To study the leakage current density performance of the symmetric MIM capacitors, an experiment on the leakage current density dependencies on the voltage (*J*–*V*) for capacitors A and B was performed. Figure 5 shows the experimental results for the two capacitors using 4200SCS. In this figure, the circle line and the triangle line represent the *J*–*V* curves of capacitor A and capacitor B, respectively. For capacitor A, the current density keeps almost constant from 0 V to 17.5 V, and then increases from 1.2 × 10^−7^ A/cm^2^ to 5.7 × 10^−6^ A/cm^2^, followed by a sharp increase to 10^−2^ A/cm^2^ at 23.8 V. For capacitor B, the current density varies very little from 0 to 22.5 V and then increases rapidly from 8.9 × 10^−8^ A/cm^2^ to 4.0 × 10^−4^ A/cm^2^ at 22.5 V and sharply to 10^−2^ A/cm^2^ at 34.1 V. It is found that the *J*–*V* curves of both capacitors A and B are divided into two regions. The variation of the *J*–*V* curves may be due to different conduction mechanisms for the MIM capacitors at low and high electric fields, which will be discussed later.

From the *J*–*V* curves in Figure 5, it is demonstrated that dielectric breakdown occurs at 23.8 V and 34.1 V for capacitor A and capacitor B, respectively. According to the electric field strength equation of *E* = *U/d*, the obtained breakdown field strength of both capacitors is 6.1 ± 0.1 MV/cm. The high breakdown strength generally enables capacitors to have a large and stable working voltage and reflects the MIM capacitor’s lifetime [2], which is strongly dependent on time-dependent dielectric breakdown (TDDB), where accelerated voltage tests are carried out to stress the capacitor at different voltages lower than the breakdown voltage for long times [21]. Also, according to the energy density equation *W* = *CU*^2^/2, the largest energy density per unit planar area of capacitor A and capacitor B is 1.23 and 1.84 mJ/cm^2^, respectively. The values of the energy density are qualified especially for a secondary power supply application.

Moreover, it is noticeable that the two capacitors have a leakage current density of approximately 10^−7^ A/cm^2^ at the low electric field. This leakage current density can meet the requirement for high density capacitors [9], shown as the red dotted line in Figure 5, and is competent for a secondary power supply application.

### 3.4. Leakage-Current-Conduction Mechanism

**Figure 6:**
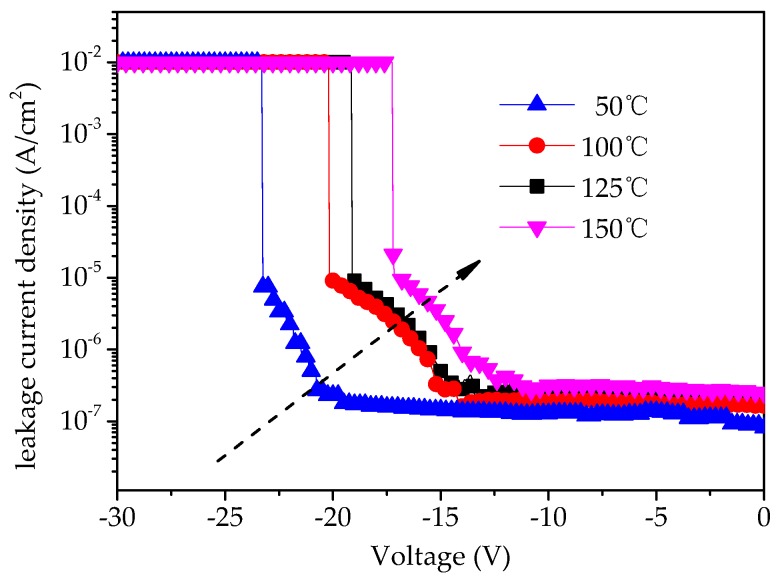
Measured leakage current density at different temperatures for capacitor A.

Figure 6 shows the measured leakage current density at different temperatures for capacitor A, which is taken as an example. It is found that the leakage current increased with increasing temperatures from 50 °C to 150 °C. The significant temperature dependence of the *J*–*V* characteristics suggests that the Schottky and Poole–Frenkel (PF) conduction mechanisms may be responsible for the obtained data.

To further understand the conduction mechanism of MIM capacitors at low and high electric field strengths, the *J*–*V* data of capacitor A was fitted with the two important conduction models of Poole–Frenkel emission and Schottky emission, which are shown as Equations (3) and (4), respectively.

(3) $$J=CE\exp\left[-\left(q\phi_{PF}-\beta_{PF}E^{1/2}\right)/kT\right]$$

(4) $$J=AT^{2}\exp\left[-\left(q\phi_{s}-\beta_{s}E^{1/2}\right)/kT\right]$$

where C and A are constants, *E* is the electric field, *T* = 298 K, *q* is the electron charge, *ϕ_PF_* is the trap height in the dielectric for PF emission [15], and *ϕ_S_* is the barrier height of the interface between the dielectric and the injecting electrode for Schottky emission, *k* is the Boltzmann constant, *β_PF_* and *β_S_* are (*q*^3^/*πε*_0_*ε_r,OP_*)^1/2^and (*q*^3^/4*πε*_0_*ε_r,OP_*)^1/2^, respectively, in which *ε*_0_ is the permittivity in vacuum, and *ε_r,OP_* denotes the dynamic permittivity measured in the optical domain (square of the refractive index, *n*^2^).

**Figure 7:**
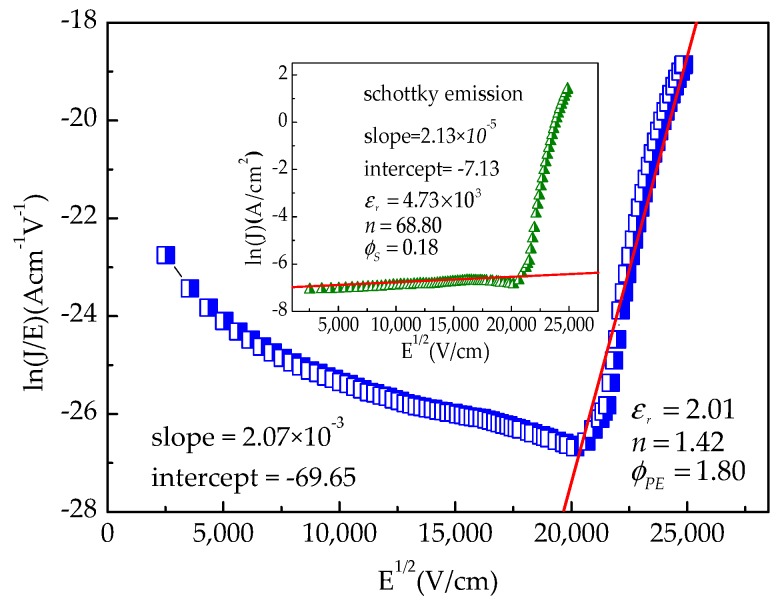
ln(*J*/*E*) versus *E*^1/2^ for capacitor A; Inset: ln(*J*) versus *E*^1/2^ for capacitor A.

Figure 7 shows the plot of ln(*J*/*E*) versus *E*^1/2^ of capacitor A according to the PF emission. It is found that the plot can be well-fitted by a straight line in the high electric field region. From the slope of the fitted line, the extracted *n* is 1.42, which is close to the reported value of 1.61 [24]. This indicates that the PF emission dominates the conduction mechanism of the MIM capacitor with an Al_2_O_3_ dielectric at high electric field regions, which is in line with that reported in Ref. [2].

The inset in Figure 7 shows the plot of ln(*J*) versus *E*^1/2^ for the Schottky emission in the low electric field. It is observed that *ϕ_S_* is 0.18 eV, which is far less than the theoretical Al_2_O_3_/TiN barrier height of 3.8 eV [14]. It indicates the presence of many interface states in the oxide, which modulate the value of the barrier height [5,7]. Also, the deduced *n* value is 68.80, which deviates severely from the aforementioned theoretical one. The result implies that no Schottky emission is present for the Al_2_O_3_ capacitor in the lower electric field.

It is a fact that neutral electron traps in oxide can generate when the electrical field is stressed on the oxide [25]. Especially, within high-*k* materials, there are more traps [1,3]. Moreover, thick oxide increases tunnelling distance but contains a large trap density, which can cause an increase in electrical stress-induced leakage current. Hence, the leakage current in the low electric field region is ascribed to trap-assisted tunnelling.

### 3.5. C–V Characteristics

**Figure 8:**
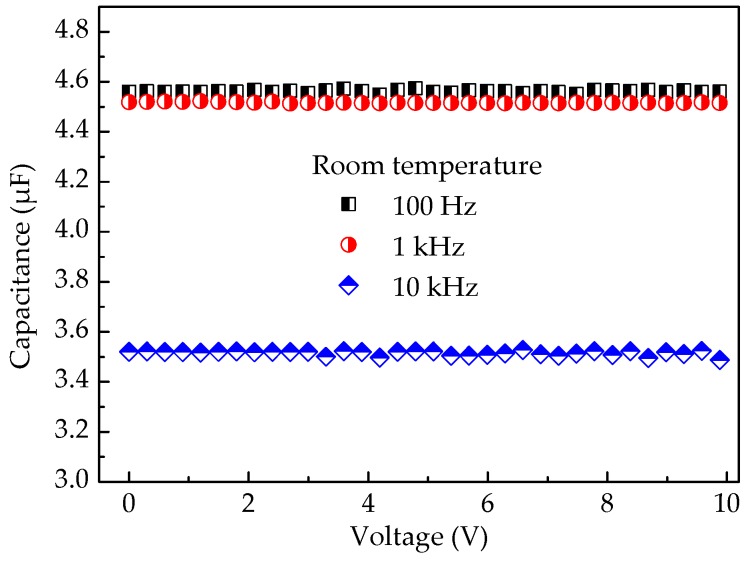
Voltage dependence on capacitance for capacitor A.

Figure 8 shows the variation of capacitance with applied voltage for capacitor A at different frequencies. It is observed that the capacitance remains nearly constant at the fixed applied frequency, which indicates the capacitor’s stability under a continuously increasing voltage stress.

**Figure 9:**
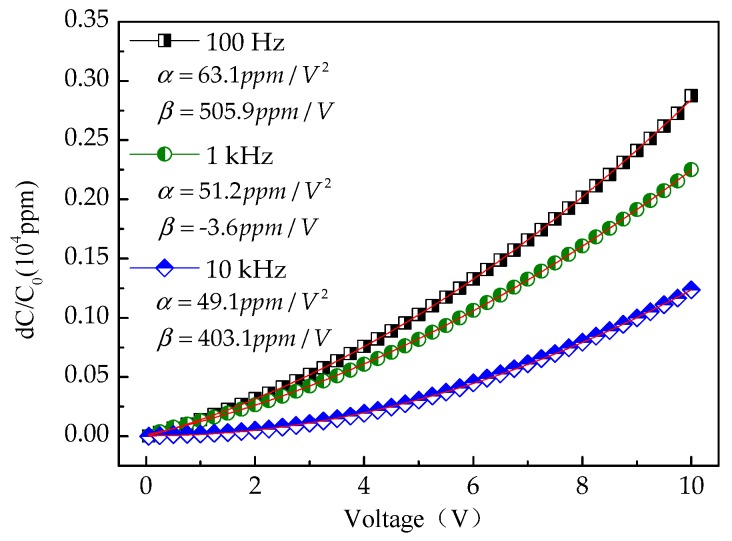
Normalized capacitance as a function of voltage of capacitor A at different frequencies.

Further, the *C–V* characteristics can be evaluated using the normalized capacitance expressed by the voltage coefficients of capacitance (VCC), which can be fitted with the following polynomial equation [9]:

(5) $$C\left(V\right)=C_{0}\left(\alpha V^{2}+\beta V+1\right)$$

(6) $$\Rightarrow \frac{C\left(V\right)-C_{0}}{C_{0}}\times 10^{6}={\left[\alpha V^{2}+\beta V\right]}_{ppm}$$

where *C*_0_ is the zero-bias capacitance, *α* represents a quadratic VCC and is driven by the application of MIM capacitors to radio-frequency circuits, and *β* is the linear VCC and demonstrates the balance of the capacitance [3]. This fitting result is shown in Figure 9.

Figure 9 shows the plot of the normalized capacitances measured at different frequencies of capacitor A. As can be seen, the extracted α values decrease from 63.1 ppm/V^2^ to 49.1 ppm/V^2^ with increasing frequency. It is attributed to the increasing frequency resulting in a longer relaxation time and a smaller capacitance variation [2]. In addition, the *α* values are lower than 100 ppm/V^2^. The result reflects a very small capacitance change of the MIM capacitors, which indicates that the fabricated capacitors have stable storage performance.

## 4. Conclusions

In this contribution, large areal (2.45 × 10^3^ mm^2^) MIM capacitors with high aspect ratio (30:1) trenches on silicon substrates using atomic-layer-deposited Al_2_O_3_ dielectric and W/TiN electrodes for a secondary power supply have been successfully fabricated and characterized in an electrical application. The resulting capacitors yield a high energy density per unit planar area of at least 1.23 mJ/cm^2^ and a high breakdown electric field of 6.1 ± 0.1 MV/cm at the voltage of 34.1 V. Also, the capacitors show a low leakage current of about 10^−7^ A/cm^2^ at 22.5 V and a low quadratic VCC of less than 63.1 ppm/V^2^. These excellent electrical properties indicate that the fabricated capacitors have high performance and can be competent for a secondary power supply application. In our future work, sandwiched multilayer MIM capacitors will be developed to meet a much larger energy density, and the effect of the microelectromechanical systems (MEMS) process on the properties of MIM capacitors will be further investigated.
